# Comparison of Ultrasound-Guided Quadratus Lumborum Plane Block and External Oblique Intercostal Plane Block for Postoperative Analgesia After Laparoscopic Cholecystectomy: A Two-Center Randomized Controlled Trial

**DOI:** 10.3390/medicina61101838

**Published:** 2025-10-14

**Authors:** Cem Kıvılcım Kaçar, Andaç Dedeoğlu, Hülya Tosun Söner, Enes Çelik, Okan Andıç, Fatma Acil, Hakan Akelma, Osman Uzundere, Erhan Gökçek

**Affiliations:** 1 Department of Anesthesiology and Reanimation, TR HSU Diyarbakır Gazi Yaşargil TRH, 21070 Diyarbakır, Turkey; anderen77@hotmail.com (A.D.); hulyatosunsoner@hotmail.com (H.T.S.); okan_andic@hotmail.com (O.A.); acilfatma@gmail.com (F.A.); osmanuzundere@gmail.com (O.U.); gokcekerhan_44@hotmail.com (E.G.); 2 Department of Anesthesiology and Reanimation, School of Medicine, Mardin Artuklu University, 47200 Mardin, Turkey; anestezistenescelik@gmail.com (E.Ç.); hakanakelma@artuklu.edu.tr (H.A.)

**Keywords:** laparoscopic cholecystectomy, quadratus lumborum plane block, external oblique intercostal plane block, postoperative analgesia, opioid consumption

## Abstract

*Background and Objectives*: Although various regional anesthesia techniques are commonly used for laparoscopic cholecystectomy (LC), to date, no randomized controlled trial has compared the effectiveness of Quadratus Lumborum Plane Block (QLB) and External Oblique Intercostal Plane Block (EOIPB) in LC. Our aim was to compare the effectiveness of ultrasound-guided QLB and EOIPB in providing postoperative analgesia after LC. *Materials and Methods*: In this two-center, randomized controlled trial, patients undergoing LC were divided into QLB and EOIPB groups. Our primary outcome was the postoperative pain scores measured using the Numerical Rating Scale (NRS) at predetermined intervals. Secondary outcomes included opioid consumption, Riker Sedation–Agitation Scale (RSAS) score, and patient satisfaction. *Results*: The NRS pain scores at postoperative 30th minute, 4th, 12th, and 24th hours were significantly lower in the QLB group (*p* < 0.05). Patients in the QLB group required significantly less tramadol compared to the EOIPB group (*p* < 0.000). The QLB group also demonstrated lower RSAS scores (*p* = 0.005), indicating a smoother recovery process. Patient satisfaction scores were markedly higher in the QLB group (*p* < 0.000). Although both blocks were well-tolerated with no differences in side effects, EOIPB was associated with higher opioid consumption, indicating its relatively limited effectiveness. *Conclusions*: To conclude, this study highlights that QLB is a more effective option for postoperative analgesia and improves patient satisfaction after LC. EOIPB may serve as a viable alternative for some patients; however, given the advantages of QLB in pain control and recovery, it stands out as a more preferable method.

## 1. Introduction

Laparoscopic cholecystectomy (LC) is known to cause less pain compared to open cholecystectomy. However, postoperative pain of varying intensities may still develop due to abdominal tension caused by pneumoperitoneum, surgical intervention, and patient-related factors [1,2]. Inadequate postoperative pain management can lead to negative outcomes such as prolonged hospital stays, increased morbidity, and higher treatment costs [3].

As components of the recommended multimodal analgesia strategy for postoperative pain management following LC, non-opioid systemic analgesics, as well as opioids, local anesthetic (LA) infiltrations (for peritoneum and skin incisions), epidural blocks, and fascial plane blocks, are commonly utilized [2,4,5]. Opioids, however, can cause side effects such as nausea, vomiting, sedation, ileus, and prolonged postoperative hospital stays [6]. With the increasing use of ultrasonography (USG), ultrasound-guided trunk blocks have gained popularity for reducing pain and minimizing opioid requirements following LC [7]. Ultrasound technology not only facilitates the application of nerve and interfascial blocks but also enables the identification and adoption of new types of interfascial blocks [1].

Ultrasound-guided quadratus lumborum block (QLB) has been demonstrated to extend to the paravertebral space due to the anatomical structure when applied between the QL muscle and the medial leaf of the thoracolumbar fascia. It provides good analgesia and provides better relief from pain associated with abdominal surgeries [2]. QLB can be performed in lateral, posterior, and anterior (transmuscular) approaches [2,3]. Recently, the anterior (transmuscular) QLB (QLB3) has gained popularity due to its effective analgesic performance in abdominal surgeries or hip arthroplasty procedures [8].

External oblique intercostal plane block (EOIPB) is a new block that has been described as an important modification of fascial plane blocks that can continuously include the upper lateral abdominal walls [9,10]. In patients undergoing this block, consistent dermatomal sensory blockade has been observed between T6-T10 at the anterior axillary line and T6-T9 at the midline. This has demonstrated its potential use for upper abdominal wall analgesia in clinical settings [10,11]. Unlike QLB, EOIPB can be performed with the patient in the supine position, offering a distinct advantage [12].

Although various regional anesthesia techniques are commonly used for LC, to date, no randomized controlled trial has compared the effectiveness of QLB and EOIPB in LC. Our primary aim is to compare the postoperative analgesic efficacy of ultrasound-guided QLB and EOIPB in LC procedures. The secondary aims include comparing these groups in terms of Riker Agitation-Sedation Scale (RASS) scores, postoperative complications, postoperative opioid consumption, and patient satisfaction.

## 2. Materials and Methods

### 2.1. Study Design, Population, and Data

The research followed the Consolidated Standards of Reporting Trials guidelines. This prospective, randomized, controlled study was conducted after obtaining ethical approval from the local ethics committee. The trial was registered at https://clinicaltrials.gov/study/NCT06666231 (date of registration: 29 October 2024) before the first patient was enrolled. All patients participating in the study received verbal information and signed an informed consent form. The study was meticulously planned in accordance with the ethical guidelines outlined in the 2013 Declaration of Helsinki.

Our study included patients aged 18–65 years who underwent LC performed by general surgery clinics at Mardin Artuklu University and Diyarbakır Gazi Yaşargil TRH in November 2024, meeting the ASA I-II risk classification criteria. Patients who declined to participate, had infections at the injection site, allergies to local anesthetics, coagulopathies, morbid obesity (body mass index [BMI] > 35), a history of drug allergies, chronic pain, long-term opioid use, psychiatric disorders, or required emergency operations were excluded from the study.

Patients were randomly allocated to either Group 1 or Group 2 through a computerized random number generator, and the assignments were secured within sealed and numbered envelopes.

Group 1: Patients who underwent Quadratus Lumborum Plane Block.

Group 2: Patients who underwent External Oblique Intercostal Plane Block.

An investigator, who had no extra tasks to do in the trial, opened the sealed envelope. Both outcome assessors and patients remained unaware of the group assignments. An anesthesiologist who was not involved in data collection or analysis performed the blocks.

### 2.2. Procedure

#### 2.2.1. Preoperative Period

Patients were evaluated preoperatively in the ward by an experienced anesthesiologist. Demographic data such as age, gender, body mass index, ASA risk classification value, and the presence of comorbidities were recorded. Detailed information was provided about the surgical procedure, anesthesia, and block management for analgesia. All patients provided informed written consent. Patients were informed about the NRS used to evaluate postoperative pain. For the NRS score evaluation, pain intensity was evaluated on a scale from 0 to 10, with 0 indicating no pain and 10 representing the most severe pain.

#### 2.2.2. Anesthesia Induction and Maintenance

On the day of surgery, after an 8 h preoperative fasting period, patients were brought to the operating room. Monitoring was conducted in accordance with ASA standards, including electrocardiography, peripheral oxygen saturation, and non-invasive blood pressure measurements. Two venous lines were opened with a 20 G cannula from the antecubital region. For anesthesia induction, patients received the following intravenously: 0.1 mg/kg midazolam (Zolamid^®^, Vem İlaç Sanayi ve Tic. A.Ş., Ankara, Turkey), 2–3 mg/kg propofol (Propofol^®^ 2% Fresenius^®^, Fresenius Kabi, Bad Homburg, Germany), 2 µg/kg fentanyl (Talinat^®^, Vem İlaç Sanayi ve Tic. A.Ş., Ankara, Turkey), and 0.6 mg/kg rocuronium (Curon^®^, Mustafa Nevzat İlaç Sanayi A.Ş., Istanbul, Turkey). After achieving adequate muscle relaxation, patients were intubated with an appropriately sized endotracheal tube. To maintain general anesthesia, a mixture of 50% oxygen and air containing 1 MAC concentration of sevoflurane (Sevorane^®^ Liquid 100%, AbbVie, Queenborough, Kent, UK) was administered at a flow rate of 3 L/min. During surgery, if the mean arterial pressure increased by 20% or more, additional analgesia was provided with 1 µg/kg intravenous fentanyl, and this was recorded.

#### 2.2.3. Block Procedure

The blocks were performed using a sterile technique under ultrasound guidance by an anesthesiologist with at least 5 years of experience. Fascial blocks were performed after endotracheal intubation. USG device (GE LOGIQ™ e, GE Healthcare, Wauwatosa, WI, USA), 20 G 100 mm short beveled block needles (Stimuplex Ultra 360, B Braun Medical, Melsungen, Germany), and the total dose and concentration of bupivacaine (Bupivacaine HCL, Buvasin 0.5%, Istanbul) were the same for all participants. Nerve blocks were performed using 20 mL on each side, a total of 40 mL of 0.25% bupivacaine. During the administration of nerve blocks, the screen of the USG device was consistently positioned away from the patient’s field of view.

##### Group 1 (QLB)

With the patients in the supine position, a lateral tilt was applied. The anterior (transmuscular) quadratus lumborum block (QLB3) method was performed. The 1–5 MHz convex ultrasound probe was placed transversely on the patient’s side, just cranial to the iliac crest and angled caudally. The goal was to visualize the acoustic shadow of the L4 transverse process, the erector spinae muscles posteriorly, the quadratus lumborum (QL) muscle laterally, and the psoas major muscle anteriorly (the “shamrock sign”).

Using an in-plane approach, the block needle was inserted from the posterior side, passing through the erector spinae and QL muscles until it reached the interface between the quadratus lumborum and psoas major muscles. The spread of 1–2 mL of LA was observed along this fascial plane. Once the needle tip’s position was verified, bupivacaine was administered into the fascial plane between the two muscles, and its spread was observed.

##### Grup 2 (EOIPB)

While the patient remained in a supine position following endotracheal intubation, a 4–13 MHz linear USG probe was placed sagittally between the midclavicular and anterior axillary lines, aligned with the sixth rib. The block needle was advanced into the space between the external oblique and intercostal muscles at the level of T6–T7. The spread of 1–2 mL of LA was observed along this fascial plane. Once the needle tip’s position was verified, bupivacaine was administered into the fascial plane between the two muscles, and its spread was observed.

#### 2.2.4. Final Stage of the Operation

Half an hour prior to the completion of surgery, every patient was administered 100 mg of intravenous tramadol (Contramal^®^, Abdi İbrahim İlaç San. ve Tic. A.Ş., Istanbul, Turkey) for analgesia and 0.1 mg/kg intravenous ondansetron (Emeset^®^, Koçak Farma İlaç ve Kimya Sanayi A.Ş., Istanbul, Turkey) to avoid postoperative nausea and vomiting (PONV). After the surgery was completed, all patients were administered 0.01 mg/kg intravenous atropine sulfate (Biosel^®^, Osel İlaç Sanayi ve Tic. A.Ş., Istanbul, Turkey) and 0.02 mg/kg intravenous neostigmine methylsulfate (Neostigmine^®^, Adeka İlaç Sanayi ve Ticaret A.Ş., Samsun, Turkey) to reverse neuromuscular blockade. The patients were then extubated. After extubation, patients’ agitation levels were assessed using the Riker Sedation–Agitation Scale (SAS) until their discharge from the Post-Anesthesia Care Unit (PACU).

#### 2.2.5. Postoperative Period

The durations of both anesthesia and surgery were noted, and the patients were admitted to the postoperative recovery unit (PACU). The NRS was used to evaluate postoperative analgesic effectiveness. For the NRS score evaluation, pain intensity was evaluated on a scale from 0 to 10, with 0 indicating no pain and 10 representing the most severe pain. NRS scores were recorded at the postoperative 30th minute and the 2nd, 4th, 12th, and 24th hours.

All patients received 1000 mg of intravenous paracetamol (Parol^®^, Atabay Kimya San. ve Tic. A.S., İstanbul, Turkey) at 8 h intervals as routine postoperative analgesia. If patients had an NRS score of ≥5, 100 mg of intravenous tramadol was administered and recorded. If no response was observed within 30 min, an additional 100 mg of tramadol was given as a rescue dose. The total amount of tramadol consumed during the first 24 h postoperatively was recorded.

Patients in the block group were closely monitored for complications such as bradycardia, hypotension, nausea–vomiting, and respiratory depression. At the end of 24 h, patient satisfaction scores were evaluated (1: Very unsatisfied, 2: Quite unsatisfied, 3: Moderate, 4: Quite satisfied, 5: Very satisfied).

### 2.3. Statistical Analysis

G-Power software (version 3.1.9.4; Kiel University, Kiel, Germany) was used to calculate the required sample size. The minimum number of patients required was found to be 48 (24 in group 1 and 24 in group 2) assuming a two-sided alpha error of 0.05, a power of 0.90, an allocation ratio of N2/N1 = 1, and an effect size of 0.99 (Table 1) [13,14].

SPSS 16.0 for Windows was used for statistical analyses. Descriptive statistics were used to express the numerical data as mean and standard deviation or median and interquartile range, while categorical data were presented as frequency and percentage. Comparison of categorical data between groups was performed using chi-square and Fischer’s exact tests, with results given as n%. The Kolmogorov–Smirnov test was used to evaluate whether non-categorical data followed a normal distribution. Student’s *t*-test was used to compare data that conformed to normal distribution, and the Mann–Whitney U test was used for data that did not conform to normal distribution. In all analyses, *p* < 0.05 was considered significant. To reduce the risk of type I error inflation due to multiple testing, the Bonferroni correction was applied. Accordingly, the adjusted significance threshold was set at *p* < 0.01 (0.05/5). The effect size (*r*) was calculated using the formula *r = Z/√N*, where *Z* represents the *z*-value obtained from the Mann–Whitney U test and *N* is the total sample size.

## 3. Results

**Figure 1:**
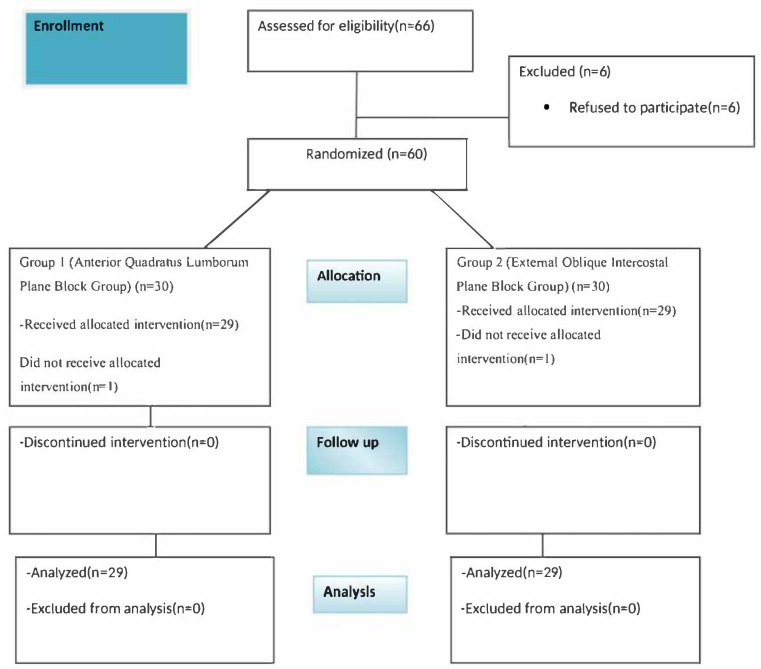
Flowchart of the study.

In total, 66 patients were assessed for eligibility, and 60 were enrolled in the study. Two patients were excluded: one in Group 1 and one in Group 2 did not receive the allocated intervention. For the remaining 58 patients, no data or measurements were missing or lost. Figure 1 presents the study flowchart.

QLB was applied to 29 patients, and EOIPB was applied to 29 patients. The mean age of the participants in the study was 43.50 ± 9.80 years. The median (IQR) values of surgery and anesthesia times were 59 (52–60.25) and 70 (68–80) minutes, in that order.

After the patients were grouped into two categories and compared according to their demographic, clinical, and intraoperative characteristics, no meaningful differences were observed between the groups.

In our study, a total of 12 patients had comorbid conditions. In Group 1, six patients (21%) presented with concomitant diseases, including hypertension in two, asthma in one, osteoarthritis in one, and diabetes mellitus in two. Similarly, six patients (21%) in Group 2 had comorbidities, comprising diabetes mellitus in two, chronic obstructive pulmonary disease (COPD) in one, and hypertension in three (Table 2).

*p*-values were adjusted for multiple comparisons using Bonferroni correction (adjusted significance threshold *p* < 0.01). After correction, significant differences in NRS scores remained at NRS1, NRS4, and NRS5, while the NRS3 difference lost significance. Median NRS scores were significantly lower in Group 1 than in Group 2 at these time points (*p* = 0.005, <0.001, and <0.001, respectively), with moderate-to-large effect sizes (*r* = 0.37–0.61). Effect sizes (*r*) were interpreted as small (≈0.1), medium (≈0.3), and large (≥0.5) [15] (Table 3).

Comparison of the groups in terms of RSAS scores following extubation revealed that Group 1 had significantly lower scores than Group 2 (*p* = 0.005) (Table 4).

Comparison of postoperative analgesic consumption showed that the total amount of tramadol administered was significantly lower in Group 1 than in Group 2 (*p* < 0.001, *r* = 0.46), indicating a small-to-moderate effect size (Table 5).

Comparison of the groups regarding postoperative patient satisfaction scores and side effects revealed that patients in Group 1 had statistically elevated satisfaction scores compared to Group 2 (*p* < 0.000). No statistically significant differences were observed between the groups concerning PONV. Additionally, no other side effects or complications were reported in either group (Table 6).

## 4. Discussion

This study compared the efficacy of ultrasound-guided QLB and EOIPB techniques for providing postoperative analgesia after LC. The findings revealed significant differences between the two block techniques regarding postoperative pain management and patient satisfaction.

In the study conducted to provide multimodal analgesia, it was observed that patients who underwent QLB had significantly lower postoperative pain scores (NRS) compared to those who underwent EOIPB. Notably, significant differences were found in NRS 1, NRS 3, NRS 4, and NRS 5 scores (*p* < 0.05). These results indicate that QLB is an effective analgesic option for laparoscopic surgeries. QLB provided better control of surgery-related pain, allowing patients to experience less pain during the postoperative period. On the other hand, EOIPB was found to have a more restricted effect on pain control.

Although LC is considered a minimally invasive procedure, most patients experience mild to moderate postoperative pain. The four trocars used in LC are placed in the subxiphoid, epigastric region, right lateral subcostal, and right subcostal-midclavicular areas. The main sources of postoperative pain are trocar entry incisions (50–70%), the effects of pneumoperitoneum (20–30%), and the cholecystectomy wound (10–20%) [16]. Management of abdominal pain following LC, especially in upper abdominal surgeries, is challenging and can impact morbidity [17]. Multimodal analgesia is widely preferred to reduce postoperative analgesic use and associated side effects [18,19]. Regional anesthesia techniques, in particular, are effective in alleviating somatic pain after LC by targeting the T6–T10 dermatomes of the intercostal nerves [20].

Ökmen et al. investigated the postoperative analgesic effects of bilateral QLB in LC procedures and observed that opioid use and pain scores were notably lower for 24 h [2]. Additionally, a review by De Cassai et al. reported that QLB effectively reduced intraoperative opioid use, postoperative pain, and PONV in patients undergoing LC [21]. Korgvee et al. highlighted that QLB offers effective postoperative pain management in abdominal surgeries, which supports the results of our study [22]. A meta-analysis demonstrated that QLB is an effective fascial block for reducing patient-reported pain and total opioid consumption after abdominal surgery [23]. In our study, we similarly found that QLB has notable postoperative analgesic efficacy in LC procedures.

The EOIPB is a more recently described technique for upper abdominal surgery [9,10]. In a cadaver study conducted in 2018, Hamilton and Manickam demonstrated that the application of local anesthetics could effectively block the lateral cutaneous branches of the T7–T11 spinal nerves [24]. Over time, Hamilton and Manickam further supported this hypothesis through a cadaver study using dyes to examine the diffusion of drugs following the EOIPB [9]. Elsharkawy and colleagues noted that EOIPB provides analgesia by blocking nerves innervating the upper midline and lateral abdominal wall. They demonstrated the potential mechanism of the block by staining the anterior and lateral branches of the T7–T10 intercostal nerves. Additionally, this block provides a dermatomal sensory block in the anterior axillary region at the T6–T10 level and in the midline at the T6–T9 level [10]. In a recent study highlighting the efficacy of EOIPB, patients undergoing laparoscopic sleeve gastrectomy who received EOIPB were compared with those who did not. The morphine consumption for 24 h was reduced by 46.4% in the EOIPB group, revealing its opioid-sparing effect [25]. In another study, the authors compared bilateral EOIPB with port-site infiltration. In comparison to port-site infiltration, lower opioid consumption and reduced pain scores were observed with EOIPB without an increase in side effects [26]. Clinical experiences with EOIPB in open nephrectomies and liver surgeries (both open and laparoscopic) have also been encouraging [27,28,29]. Ultrasound-guided nerve blocks reduce the risk of local anesthetic systemic toxicity (LAST). Nevertheless, lipid emulsion should be readily available to manage unexpected cases [30].

In the postoperative period, when examining the patients’ comfort during the anesthesia recovery process, the group that received QLB had significantly lower RSAS scores (*p* = 0.005). This suggests that QLB provides a more stable awakening process and requires less sedation post-anesthesia. On the other hand, the higher RSAS scores in the EOIPB group indicate that this block technique may be associated with an increased need for sedation and anesthesia. The effect of QLB on sedation has also been reported in earlier studies. As an example, studies by Uppal et al. and Liu et al. have shown that QLB improves patients’ anesthesia recovery and leads to faster recovery [31,32].

In our study, the QLB group showed a significant reduction in the amount of tramadol used for postoperative analgesia (*p* < 0.000). This indicates that QLB provides effective analgesic effects and reduces the need for additional analgesic medication. In contrast, the higher tramadol consumption in the EOIPB group suggests that the pain-relieving effect of this method is limited. Reducing the use of analgesics is an important factor in improving patient comfort during the treatment process. The analgesic efficacy of QLB has been documented in various earlier studies on abdominal surgeries. In particular, Liu et al. found that QLB improved postoperative analgesia and reduced the use of analgesic drugs in abdominal surgeries [32]. Additionally, Akerman et al. reported that QLB significantly reduced postoperative pain and decreased medication requirements, especially in laparoscopic surgeries [33]. Another systematic review found that QLB reduced cumulative 24 h opioid consumption following abdominal surgical operations [34]. Higher tramadol use in the EOIPB group indicates that this technique has limited pain management capacity. Reducing analgesic use is a key factor in ensuring that patients experience a smoother recovery, and this serves as a significant advantage of QLB in clinical practice. Both Korgvee et al. and Hassanein et al. emphasize that QLB increases analgesic efficacy and significantly reduces the need for analgesic drugs such as tramadol [22,35].

When we assessed patient satisfaction, we observed that the QLB group had significantly higher satisfaction scores compared to the EOIPB group (*p* < 0.000). This supports the positive impact of QLB on patients’ overall well-being. Similarly, Korgvee et al. reported that QLB enhances patient satisfaction [22]. Additionally, our study revealed no significant differences between the two groups regarding PONV or other side effects. This effect could be linked to the use of ondansetron. Consequently, both block techniques appear to be safe and well-tolerated methods.

Our study has certain limitations. The limited number of patients reduces the generalizability of the results. Additionally, only patients undergoing laparoscopic cholecystectomy (LC) were included; thus, additional studies are necessary to assess the effectiveness of QLB and EOIPB in different surgical procedures. Prolonged post-procedure data are insufficient, and larger studies are necessary to assess the long-term effectiveness and side effects of these block techniques. Moreover, pain scores are subjective and may vary due to individual differences. The absence of deep anesthesia monitoring represents another limitation of this study, as it might have influenced the findings related to sedation assessed by the RSAS.

## 5. Conclusions

To conclude, this study highlights that QLB is a more effective option for postoperative analgesia and improves patient satisfaction after LC. EOIPB may serve as a viable alternative for some patients; however, given the advantages of QLB in pain control and recovery, it stands out as a more preferable method. These findings suggest that QLB could play a significant role in improving postoperative analgesia strategies for laparoscopic surgeries.

## Figures and Tables

**Table 1:** Power analysis standard table [13,14].

We performed the POWER analysis	Before starting
On the primary outcome	NRS
Based on the two-tailed statistical test:	Two-tailed
And accepting the cutoff for significance (*α*)	0.05
And a power (1 − *β*) of	0.90
The variability of the primary outcome was	In the study by Özdemir et al., 2 h NRS scores were taken into account (mean + SD for group 1 = 1.4 ± 0.9 and for group 2 = 2.4 ± 1.1) [14]
Consequently, the effect size was	0.99
The total sample size needed was	48

**Table 2:** Comparison of patients’ demographic, clinical, and intraoperative characteristics.

Characteristic	All Patients (N= 58)	Group 1 (n = 29)	Group 2 (n = 29)	*p*-Value
	Mean ± SD	Mean ± SD	Mean ± SD	
Age (years)	43.50 ± 9.80	42.83 ± 8.12	44.17 ± 11.33	0.60
BMI (kg/m^2^)	28.02 ± 3.63	27.68 ± 3.35	28.36 ± 3.92	0.47
	Median (IQR)	Median (IQR)	Median (IQR)	
Surgery duration (min)	59 (52–60.25)	60 (55–60)	56 (51–62)	0.53
Anesthesia duration (min)	70 (68–80)	70 (70–80)	70 (64–79)	0.13
	n (%)	n (%)	n (%)	
Gender				0.26
Male	20 (%)	12 (%41)	8 (%27)	
Female	38 (%)	17 (%59)	21 (%73)	
Comorbidity				1
Yes	12 (%21)	6 (%21)	6 (%21)	
No	46 (%79)	23 (%79)	23 (%79)	
ASA				0.59
I	28 (%48)	15 (%52)	13 (%45)	
II	30 (%52)	14 (%48)	16 (%55)	

BMI: Body mass index; ASA: American Society of Anesthesiology.

**Table 3:** Comparison of groups based on numerical rating scale (NRS) scores.

	Group 1 (n = 29)	Group 2 (n = 29)	Effect Size (*r*)	*p*-Value	Bonferroni *α* = 0.01
	Median (IQR)	Median (IQR)			
NRS1	2 (1–2)	3 (2–4)	0.37 (medium)	0.005	Significant
NRS2	2 (2–2.5)	2 (2–3.5)	0.23 (small)	0.074	NS
NRS3	2 (1–2)	2 (1–3.5)	0.29 (small-medium)	0.028	NS
NRS4	1 (1–2)	3 (2–5)	0.61 (large)	<0.000	Significant
NRS5	1 (1–1)	2 (1.5–3)	0.52 (large)	<0.000	Significant

NRS1: Postoperative 30th minute, NRS2: Postoperative 2nd hour, NRS3: Postoperative 4th hour, NRS4: Postoperative 12th hour, NRS5: Postoperative 24th hour.

**Table 4:** Comparison of groups based on RSAS scores.

	Group 1 (n = 29)	Group 2 (n = 29)	*p*-Value
	n (%)	n (%)	
RSAS Score			0.005
1 Unarousable	0 (%)	0 (%0)	
2 Very sedated	4 (%)	0 (%)	
3 Sedated	12 (%)	8 (%)	
4 Calm and cooperative	9 (%)	9 (%)	
5 Agitated	4 (%)	10 (%)	
6 Very agitated	0 (%)	2 (%)	
7 Dangerously agitated	0 (%0)	0 (%0)	

SAS: Sedation–Agitation Scale.

**Table 5:** Comparison of groups based on the amount of tramadol used in the first 24 h postoperatively.

	Group 1 (n = 29)	Group 2 (n = 29)	Effect Size (*r*)	*p*-Value
	Median (IQR)	Median (IQR)		
Amount of Tramadol Used (mg)	0 (0–0)	0 (0–100)	0.46 (small-medium)	<0.000

**Table 6:** Comparison of the groups in terms of patient satisfaction score and nausea–vomiting.

	Group 1 (n = 29)	Group 2 (n = 29)	*p*-Value
	n (%)	n (%)	
Patient Satisfaction Score			<0.000
Very dissatisfied	0 (%)	0 (%)	
Somewhat dissatisfied	0 (%)	7 (%)	
Neutral	2 (%)	9 (%)	
Quite satisfied	13 (%)	10 (%)	
Very satisfied	14 (%)	3 (%)	
Side Effects (Nausea–Vomiting)			0.68
Yes	3 (%)	4 (%)	
No	26 (%)	25 (%)	

## Data Availability

The data that support the findings of this study are available on request from the corresponding author.
